# Knowing What Counts: Unbiased Stereology in the Non-human Primate Brain

**DOI:** 10.3791/1262

**Published:** 2009-05-14

**Authors:** Mark Burke, Shahin Zangenehpour, Peter R. Mouton, Maurice Ptito

**Affiliations:** Department of Physiology, Universite de Montreal - University of Montreal; Ecole d’optometrie, Universite de Montreal - University of Montreal; Stereology Resource Center

## Abstract

The non-human primate is an important translational species for understanding the normal function and disease processes of the human brain. Unbiased stereology, the method accepted as state-of-the-art for quantification of biological objects in tissue sections^2^, generates reliable structural data for biological features in the mammalian brain^3^. The key components of the approach are unbiased (systematic-random) sampling of anatomically defined structures (reference spaces), combined with quantification of cell numbers and size, fiber and capillary lengths, surface areas, regional volumes and spatial distributions of biological objects within the reference space^4^. Among the advantages of these stereological approaches over previous methods is the avoidance of all known sources of systematic (non-random) error arising from faulty assumptions and non-verifiable models. This study documents a biological application of computerized stereology to estimate the total neuronal population in the frontal cortex of the vervet monkey brain (*Chlorocebus aethiops sabeus*), with assistance from two commercially available stereology programs, BioQuant Life Sciences and Stereologer (Figure 1). In addition to contrast and comparison of results from both the BioQuant and *Stereologer* systems, this study provides a detailed protocol for the *Stereologer* system.

**Figure:**
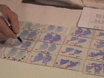


## Protocol

### Part 1: Pre-processing of tissue should be done according to Burke et al. (2009)^5^.

Briefly, tissue should be well 	perfused with paraformaldehyde, glutaraldehyde, or formalin. This 	can be achieved through standard transcardial perfusion typically 	used to harvest other organs. In the present study the subject was 	deeply sedated with ketamine hydrochloride (10 mg/kg, i.m.), 	euthanized with an overdose of sodium pentobarbital (25 mg/kg, i.v.) 	and perfused transcardially with 0.1 M PBS until completely 	exsanguinated. This is followed by a 4% paraformaldehyde solution in 	PBS for 5 min (~1 liter). Tissue should be well perfused with 	paraformaldeyhde, gluteraldehyde, or formalin. The brain should be 	stereotaxically blocked, removed from the skull, weighed, volume 	determined, cryoprotected, and frozen^5^.

### Part 2: Systematic Sampling of tissue, according to Burke et al. (2009)^6^.

In this example we focused on the 	frontal lobe of the vervet brain. The reference space was defined to 	include cortical tissue from the central sulcus to the frontal pole 	of the left hemisphere. For our purposes the series were set at 1/10 	sections throughout the cortex, sectioned at 50µm. One complete 	series was stained with cresyl violet (series #1). Series 2 was 	split so that half of the sections were stained with gold chloride 	for myelin revelation and the other half stained for acetylcholine 	esterase (for delineation of certain subcortical areas). All other 	series were banked in antigen preserve as part of our long-term 	research plans.

### Part 3: Stereology (for more details, see Mouton 2002)^4^

The total estimation of cell 	numbers (*N*) is calculated based on the following equation: *N* = *ssf*-1 x *asf*-1 x *tsf*-1 x Σ*Q*- Where *ssf* is the section sampling fraction, *asf* is the area sampling fraction, *tsf* if the thickness sampling fraction (where the measured thickness of the tissue is divided by the dissector height), and ∑*Q*- is the total number of objects of interest counted within the dissector. The next sections of this protocol show how to determine the *ssf*, *asf*, *tsf* and ∑*Q*.
Section Sampling Fraction (non-computer based)
  The entire reference space must be well defined from anterior to posterior and dorsal to ventral. In this example we were interested in the frontal lobe and have defined it as the region from the tip of the frontal pole (anterior) to the central sulcus (posterior) and the lateral sulcus (ventral) and excluded the insula.
  For the frontal lobe of this particular subject a total of 760 sections were systematically collected with 76 stained for cresyl violet. Since the target was 10 sections throughout the reference space 1/6 cresyl violet stained sections were sampled, leading to a Section Sample Fraction of about 1/60. We randomly started with one of the first 6 cresyl violet stained sections and systematically sampled 1/6 thereafter. A total of 13 sections were sampled for the selected subject (Figure 2).
Area Sample Fraction 	(computer-based)
  A pilot study will indicate the optimal grid size, the Area Sampling Fraction, to sample the reference space with about 100-300 disectors. Within the frontal lobe, a grid size of 2500 µm^2^ yielded an average of 254 disectors for this subject (Figure 2). The size of the disector should yield between about 0-5 counted objects, in this case, neurons.
Thickness Sample Fraction 	(computer-based)
  At each disector location, the height of the tissue is measured, and a fraction of this height is sampled; this is known as the Thickness Sampling Fraction. To determine the measured thickness, focus through the z-plane until the first cell comes into focus, then back up slightly to the top of the section, i.e., until the last object appears just out of focus. To determine the bottom of the tissue, focus through the z-plane until the cells are barely out of focus.
  Focusing through the z-plane, cells should be stained at every depth; if not, this may indicate an incomplete penetration of the stain or non-uniform dehydration of the tissue, in which case the sections should be re-stained. This precaution is especially important for immunostained tissue that requires penetration of immunoprobes through relatively thick tissue sections. For this reason, immunostained tissue should be lightly counterstained with a basophilic stain (e.g., cresyl violet, hematoxylin) in order to accurately determine top and bottom of the section and to confirm penetration of the antibody. In this study the sections were sliced at a microtome setting of 50µm. After all tissue processing was complete, the measured tissue thickness averaged 17.9µm (Figure 2), with a mean shrinkage of about 65%. Note that these results represent typical shrinkage for tissue processed for routine histological preparation^7^.
  The default disector height for the typical study is 10µm. The difference between the disector height and the measured section thickness is the guard height, the volume of tissue where no biological features are counted. The use of a guard height avoids tissue damage at the sectioning surfaces (e.g., lost caps).
The total number of objects 	counted, Σ*Q*-
  To avoid bias from recognition errors, it is imperative that a standard definition is followed for the particular biological feature of interest. In this case, we were interested in counting neurons. Therefore, a neuron was defined as having a visible centrally located nucleolus and a clearly defined cytoplasm, whereas glial cells generally lacked visible nucleoli and cytoplasm. For this subject 457 neurons were counted.

### Part 4: *Stereologer* -- A Computerized Stereology System (Stereology Resource Center, Chester, MD)

The *Stereologer* System prompts the user to fill out the necessary information in a step-by-step fashion. In the “Study Information” section the 	parameters of the study are established. Since a reference space must be defined for the study, the volume parameter should be 	selected (for the reference space the Cavalieri estimator should be selected). Object volume may also be selected here to estimate the 	number-weighted volume for the population of objects of interest. 	For our example, only the volume of the reference space was selected. For each object, select the number and define the feature of interest, in this case Neurons.
Case Initialization: In this 	section the sampling information is established. Enter the slab sampling interval (if separate slabs of tissue were sliced 	exhaustively and each section is placed in sequential order then enter 1 here). Enter the total number of sections taken through the reference space (in this case 760). Then enter the section sampling interval (in this case 59). The system then calculates the number of sections to be sampled (in this case 13).
Probe Parameters: This section 	defines the grid and disector size. For the volume, use a low 	magnification 2.5x-10x to define the grid spacing under the edit 	menu. Object magnification should be performed at 100x (N.A. 1.3 or 	1.4). Frame area is the size of the disector, in this case we used 	50% Screen. The frame height is the thickness of the disector; set 	at 10µm here. The frame spacing is the size of the grid. In our 	pilot study we found that 2500µm yields between 150-200 frames 	spaced in a systematic-uniform manner through a relatively large 	reference space. For smaller areas, a smaller grid size should be 	used. A pilot study will identify the optical probe parameters for 	each particular study.
Once the study parameters have 	been established, sampling through the tissue may proceed. The 	program will prompt you to sample the first section. **Step 1:** The program will prompt the user, under low magnification, to trace 	the reference space on the section. The system will then place a 	grid over the section based on the probe parameters and the user 	will then verify that points fall within the reference space. If a 	point is not within the reference space simply click on the point 	and will not be calculated into the volume. **Step 2:** The 	system will place a new grid over the reference space, based on the 	frame spacing parameters. Verify that the intersections fall within 	the reference space. **Step 3: **The system will then prompt the 	user to switch to the higher magnification objective and move the 	stage to the first dissector. **Step 4: **At this point the 	system prompts the user to define the top and bottom of the section 	then the system sets the sampling space in the z-axis. **Step 5: **The user will then click on each object that falls within the 	disector through the z-plane. Objects that touch the red lines or 	the bottom of the disector may not be counted. Once each object is 	counted, click next. The stage will move to the next disector and 	steps 4 and 5 will be repeated. Once all of the disectors for the 	section have been counted, the system will prompt the user to insert 	the next sequential section to be probed. Steps 1-5 will be repeated 	until all of the sequential sections have been sampled. The system 	will then provide the calculations for *asf*, *ssf*, *tsf*, 	and ∑*Q*. Based on these parameters, the system will generate 	an estimated N, reference volume and CE’s for both the estimated 	number and volume. It will also give recommendations to become more 	efficient or to reduce the *CE* (Figure 2).

### Part 5: Representative Results:

Unbiased stereology provides efficient and reliable estimates of cell populations within a reference space. There are a number of computer-based stereological systems available, all of which rely on systematic sampling and a defined reference space. We have used the BioQuant Life Sciences system to estimate the total neuronal population of the cerebral cortex of 2-year-old vervets to be over 828 millions (Figure 3) with an average CE of 0.042.  We have subsequently used the *Stereologer *system to estimate that the frontal lobe accounts for about half the number of cortical neurons^8^.


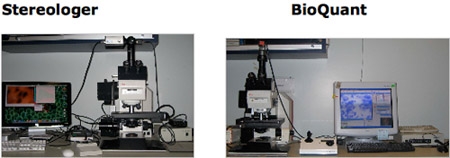
 **Figure 1 Computer-based Stereology Systems.** The basic set-up of the stereology systems is the same, a microscope with a motorized stage (x-y-z), a stage controller, video camera, computer, and software. The system that is ultimately chosen should be based on efficiency, needs, and cost analysis.

**Table:** 

STUDY INFORMATION
Study Name	Frontal Cortex
Principal Investigator	MB
Species	AG
Reference Space Frontal	Cortex
Notes
CASE INFORMATION
Data Collector	MB
Date	Wednesday, April 30, 2008
Group	Control
Subject	O26
Notes
SAMPLING CHARACTERISTICS
Slab Sampling Interval	1
Total Number of Sections	760
Section Sampling Interval	59
Starting Section	2
FRACTIONS
ASF	0.0002
SlabSF	1.0000
SSF	0.0169
TSF	0.5587

**Table:** 

RESULTS SUMMARY
Parameter	Probe	Name	Result	CE	SD
Thickness	---	---	17.8996 µ	---	---
Number	Disector	neuron	243833769.1473	0.0474	N/A
Volume	Cavalieri Point Grid	volume	1719769397954.1284 µ^3	0.0078	N/A

**Table:** 

RECOMMENDATIONS
Probe	Object Number (neuron)
CE	0.0474
Recommendations	CE is acceptable.

**Table:** 

Number of Disectors Viewed is Too High, Increase Disector Spacing.
Probe	Region Volume (volume)
CE	0.0078
Recommendations	CE is acceptable.

**Table:** 

SECTION RESULTS
Section	neuron NumObjects neuron NumCorners	volume RegPoints
1. 40	48	34
2. 26	52	44
3. 29	56	48
4. 39	100	83
5. 49	112	83
6. 53	156	120
7. 65	128	106
8. 45	108	100
9. 52	100	77
10. 26	64	60
11. 21	44	44
12. 11	36	32
13. 1	12	6

**Figure 2 Results from the Fontal Lobe Obtained with the Stereologer System.** For the frontal lobe, the longest part of the stereological process was in the systematic sampling and sectioning. Topography, volume and neuronal estimate were completed in about a day for each subject. The left hemisphere was sampled and the number is doubled in the text to approximate an estimate for both hemispheres.


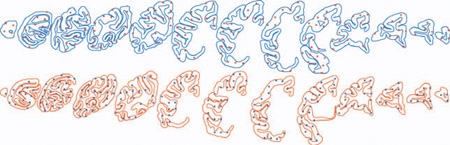
 **Figure 3 Cortex Neuronal Population Estimate Obtained with BioQuant.** The BioQuant system requires an exact topography prior to cell counting.  Here an average of 13 sections were sampled. Cortical topography and subsequent volume estimation took between 15-20 hours for each subject. Topography was performed under a 2.5x objective and counting was done using a 100x oil immersion objective (N.A. 1.3). Every 100th section was chosen throughout the left hemisphere with an average of 180 disectors sampled. Once the topography was completed neuronal counting took about a day. The left hemisphere was sampled and the number is doubled in the text to approximate an estimate for both hemispheres.

## Discussion

Unbiased stereology in a primate brain is similar to that of other biological specimens. However, given the size of the primate brain, a careful processing plan is recommended since a single hemisphere of the vervet monkey yields more than 1200 sections when sliced at 50µm. First we recommend stereotaxically blocking the brain into 1cm blocks, which provides a standard plane of section between blocks and subjects, minimizes partial sections between blocks, and provides manageable sized blocks^5^. A crucial step for stereology is systematic sampling such that 6-10 sections are obtained through the reference area. For the primate brain, this leaves a significant amount of material unprocessed, so in order to maximize the potential data obtained from each brain we suggest banking the tissue in antigen preserve for long-term research plans^6^. When tissue is banked, a systematic immunodetection approach for identifying target proteins in the monkey brain can be prepared for stereology^9^. Larger areas such as the cortex or the lobes may require 10-14 sections, so prior to sectioning a plan should be made to have at least 10 sections through the smallest reference space of potential interest. A pilot study should also be performed on a control subject to set the parameters for the stereological study. Furthermore when presenting or interpreting published stereological data the *asf*, *tsf*, *ssf*, and ∑*Q* should be reported along with the appropriate CE values.
